# Systematic Review of Adverse Effects: A Further Step towards Modernization of Acupuncture in China

**DOI:** 10.1155/2015/432467

**Published:** 2015-08-03

**Authors:** Junyi Wu, Yanmei Hu, Yin Zhu, Ping Yin, Gerhard Litscher, Shifen Xu

**Affiliations:** ^1^Acupuncture Department, Shanghai Municipal Hospital of Traditional Chinese Medicine, Shanghai 200071, China; ^2^Huadong Hospital Affiliated to Fudan University, Shanghai 200040, China; ^3^Research Unit for Complementary and Integrative Laser Medicine, Research Unit of Biomedical Engineering in Anesthesia and Intensive Care Medicine, and TCM Research Center Graz, Medical University of Graz, 8036 Graz, Austria

## Abstract

As a further step towards the modernization of acupuncture, the objective of this review was to figure out the frequency and severity of adverse complications and events in acupuncture treatment reported from 1980 to 2013 in China. All first-hand case reports of acupuncture-related complications and adverse events that could be identified in the scientific literature were reviewed and classified according to the type of complication and adverse event, circumstance of the event, and long-term patient outcome. The selected case reports were published between 1980 and 2013 in 3 databases. Relevant papers were collected and analyzed by 2 reviewers. Over the 33 years, 182 incidents were identified in 133 relevant papers. Internal organ, tissue, or nerve injury is the main complications of acupuncture especially for pneumothorax and central nervous system injury. Adverse effects also included syncope, infections, hemorrhage, allergy, burn, aphonia, hysteria, cough, thirst, fever, somnolence, and broken needles. Qualifying training of acupuncturists should be systemized and the clinical acupuncture operations should be standardized in order to effectively prevent the occurrence of acupuncture accidents, enhance the influence of acupuncture, and further popularize acupuncture to the rest of the world.

## 1. Introduction

Complications and adverse effects in medical practice are always of concern to the public and the medical profession. While being widely used in current medical treatment, especially in the area of intertrochanteric hip fractures, the cephalomedullary devices (CMN), however, led to a considerably high complication rate of 41.9% according to Pui et al. [1]. Faunø et al. [2] conducted a study on the medical records of 997 patients who were suffering from stoma closure, which revealed 131 cases of early complications and 187 late ones, accounting for 13.1% and 18.8% of the total subject pool, respectively. Umuroglu et al. [3] identified that the nuss procedure showed an overall complication rate of 18.7% through a retrospective analysis. Fortunately, with the increasing attention from the society on medical care safety and the further standardization of doctor's qualifications, such medical incidents have been decreasing. As acupuncture and moxibustion (A&M) are increasingly used in world, their widening acceptance necessitates continual safety assessment. This review, a sequel to two previous reviews from our team [4, 5], is an evaluation of the frequency and severity of adverse events (AEs) for acupuncture reported between 1980 and 2013 in China. These two reviews reported the AEs of A&M in the West, reported from 1965 to 1999 and from 2000 to 2011, respectively. In the first review, the main source of infection was found to be hepatitis caused by reused needles. In the second review, the majority of infections were bacterial, caused by skin contact at acupoint sites, without cases of hepatitis. For these two reviews, we just searched English articles, without Chinese reports, so it is unclear which AE happens in China. Since acupuncture is widely used in China, it is necessary to make sure what is happening about AE. In this review, we found internal organ, tissue, or nerve injuries were the main complications of acupuncture in China from 1980 to 2013. Clearly, guidelines must be followed in order to minimize acupuncture-related AEs and to modernize acupuncture and make it safer to apply.

## 2. Materials and Methods

### 2.1. Inclusion Criteria

All case reports concerning acupuncture-related complications or AEs in China published from 1980 to 2013 were identified. Only firsthand reports were included to avoid multiple reports of the same event. Thus, reviews, comments, or case-control studies were excluded from this review.

Complications, defined as “an added difficulty: a complex state, a disease, or an accident superimposed upon another without specific relation,” include infections, internal organ or tissue injuries, and other severe consequences. AEs and adverse reactions were defined as “development of an undesired side effect or toxicity” and include less severe consequences, such as contact dermatitis. Side effects, defined as “the action or effect other than that desired,” are included within the discussion of AEs [4, 5].

### 2.2. Search Strategy

The following databases were searched for case reports published from 1980 to 2013: VIP science and technology periodical database (CQVIP), China National Knowledge Infrastructure (CNKI), and Wanfang Database (WF). Search terms included “acupuncture, electro-acupuncture, acupuncture points, ear acupuncture, needling.” These terms were combined with “safe, safety, adverse event, adverse reaction, side effects, side events, complications, and risk.”

### 2.3. Data Extraction

**Figure 1:**
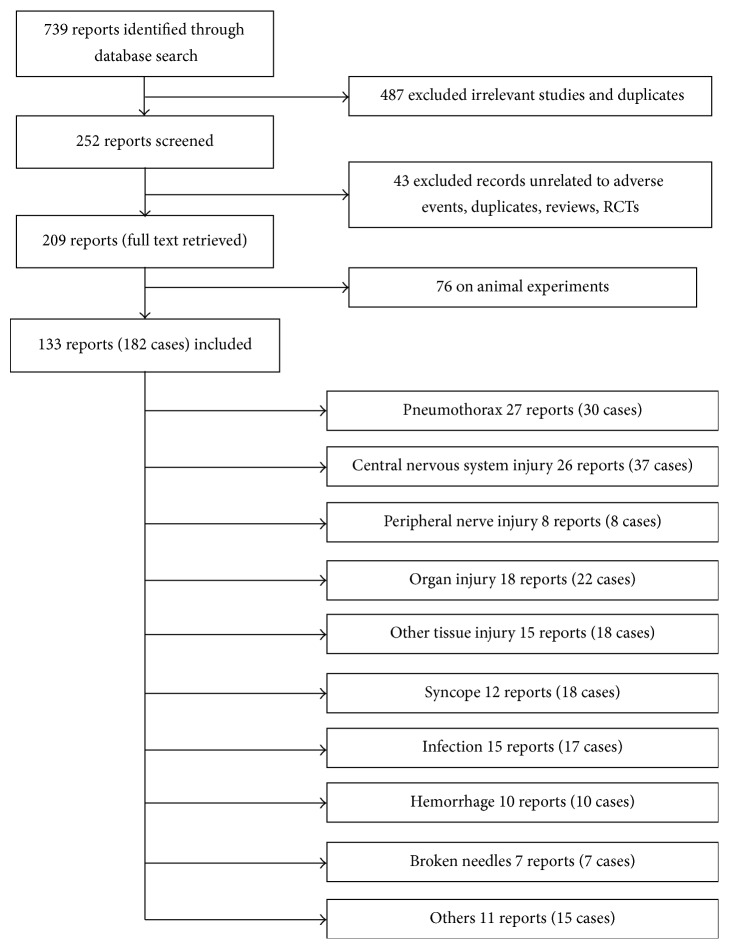
Flow chart of the screening process.

A total of 739 papers were found; 133 were relevant (Figure 1). Information pertaining to the author, year of publication, number of patients, patient's age, sex, original treatment, the punctured site, circumstances of the complication or AE, and long-term patient outcome when available was extracted from each case. These data were then organized according to the type of complication or AE.

## 3. Results

A total of 133 papers published from 1980 to 2013 were reviewed, and 182 cases of complications or AEs were identified to be associated with pneumothorax (*n* = 30), central nervous system injury (37), peripheral nerve injury (8), organ injury (22), other tissue injury(18), syncope (18), infections (17), hemorrhage (10), complications caused by broken needles (7), and others (15). Pneumothorax and central nervous system injury were the major complications.

### 3.1. Complications of Acupuncture: Internal Organ, Tissue, or Nerve Injury

A total of 115 cases of internal organ, tissue, or nerve injuries were reported, including pneumothorax (*n* = 30), central nervous system injury (37), peripheral nerve injury (8), organ injury (22), and other tissue injury (18).

### 3.2. Pneumothorax (Table 1)

Between 1980 and 2013, pneumothorax was the most common complication of acupuncture treatment, as 30 cases in 27 papers were noted to be possibly associated with acupuncture (Table 1). Among the 30 cases presented, 25 recovered through thoracocentesis, thoracic closed drainage, anti-infection treatment, and clinical observation; 2 patients died; and the outcomes of the other 3 cases were not stated or unknown. More than half of them were reported by doctors in emergency rooms or departments of internal medicine, but the practitioner's training background was not reported. The punctured sites were mostly in the chest, supraclavicular fossa, and the back. The patients' major complaints were chest stuffy, chest pain, and dyspnea. One pneumothorax patient did not receive timely treatment and died [6]. Another case died because of a tension pneumothorax after acupuncture treatment [7].

### 3.3. Central Nervous System Injury (Table 2)

There were 37 cases of central nervous system injury associated with acupuncture (Table 2). The 37 patients suffered subarachnoid hemorrhage (*n* = 27), subdural hematoma (2), spinal cord injury (2), cerebral hemorrhage reformulation (2), cervical spinal epidural hematoma (1), medulla oblongata hemorrhage (1), cisterna magna hemorrhage (1), and leukemia acute intracerebral hemorrhage (1). The causes were acupuncture of cervical acupoints (*n* = 31), acupoints between the second and third thoracic spinal process [8], acupoints KI01 (Yongquan) [9] and BL37 (Yinmen) [10], waist acupoint [11] (each *n* = 1), and body acupuncture (*n* = 2) [12, 13]. Among the 37 cases, 26 recovered and 11 died.

Because of insufficient compliance and protected observation, accidents occurred in 3 schizophrenia patients, of whom 2 died and 1 recovered [14]. Two cerebral hemorrhage patients after receiving body acupuncture died from recurrence, and the authors speculated the reason might be that acupuncture had irritated the parasympathetic nerve, causing vascular contraction and increasing blood pressure and thus cerebral hemorrhage recurred before the original cerebrovascular fracture could be fully restored [9, 12]. Two patients suffered from dizziness and vomiting during treatment, but the acupuncturists did not pay high attention; the symptoms did not improve significantly after treatment, but the acupuncturists did not realize the severity and even allowed the patients to go home. One patient was treated the next day after onset and was diagnosed as subarachnoid hemorrhage; he recovered and left hospital [11]. The other patient died the next day [13].

### 3.4. Peripheral Nerve Injury (Table 3)

There were 8 cases of acupuncture-induced peripheral nerve injury (Table 3). They include aggravated facial paralysis [15], mistakenly hitting the vagus [16], phrenic nerve injury [17], optic atrophy [18], oculomotorius injury [19], right trigeminal nerve injury [20], sciatic nerve injury [10], and peroneus nerve injury [21], respectively. The patient with optic atrophy lost sight [18], the patient with right trigeminal anchor injury improved after 3 days [20], the patient with sciatic nerve injury did not recover [10], and the other 5 patients recovered.

### 3.5. Organ Injury (Table 4)

Twenty-two cases were reported to have organ injury associated with acupuncture treatment (Table 4). There was cardiovascular injury (*n* = 7) [14, 22–27], thoracic duct injury (1) [28], peritonitis induced by abdominal system (9) [29–33], gastric perforation (3) [34–36], intestinal obstruction (1) [37], and multiple organ injury (1) [38]. Of them, 14 recovered, 7 died, and 1 did not recover. Most of them were caused by too deep puncture and incorrect acupoint location.

One patient received treatment following gastric abscess induced by gastric ulcer. He was treated by electroacupuncture (EA) at ST36 (Zusanli) and the acupoint was located without violation, but the state of illness was not relieved and gastric perforation occurred. The doctor was puzzled and put forward this case for discussion [35]. One patient with a history of stomach bleeding received treatment following knee pains induced by rheumatoid arthritis and took anti-inflammatory analgesic drugs for a long time. The lower limb acupoint was selected, but the excessive EA irritation during treatment caused an irritable gastric ulcer and then hemorrhagic shock and the patient recovered after timely rescue [36].

### 3.6. Other Tissue Injuries (Table 5)

In addition to injuries of the organs in thorax and abdomen, 18 cases of other tissue injuries were reported (Table 5), including cervical common carotid aneurysm [39], shock [38, 40], asphyxia [41], dyspnea [42], eye injury [18, 43, 44], and the locomotor system injury [10, 44–50]. Among the 18 cases, 12 recovered, 2 improved, 2 did not recover, 1 died, and the outcome of 1 was not stated.

One patient suffered from retinal detachment, and eyesight was corrected to 0.2 after treatment [43]. One patient lost eyesight because of traumatic cataract [18]. One patient experienced femoral neck fracture after strong stimulation to myospasm [45]. One patient suffered from subluxation in right wrist joint due to excessive EA intensity [48].

### 3.7. Syncope (Table 6)

A total of 18 cases of acupuncture-associated syncope were reported (Table 6). Syncope occurred during the treatment in 9 cases, several minutes after the treatment in 5 cases, and several hours later in 4 patients. Two patients fainted after taking liquors [51, 52]. The positions were sitting (*n* = 5), lying (5), and not specified (8). Most patients recovered with rest and drinking sugar water, while 2 patients recovered after injection of metoclopramide via ST36 [53]. Two cases suffered from sudden cardiac arrest and were cured after first aid [54, 55]. One patient recovered after massaging an ear acupoint [56].

### 3.8. Infection (Table 7)

A total of 17 cases were infections associated with acupuncture (Table 7). Among them, 10 recovered, 3 died, 2 improved after 3 days, 1 was disabled, and 1 was not stated. The infection was caused by tetanus bacillus (*n* = 6) [10, 57–61], hydatid (1) [62],* Escherichia coli* (1) [63], and* Mycobacterium tuberculosis* (3) [64]. One patient was infected after deep 3-degree burning [65] while others were not stated.

In one patient, the right epigastric mass due to acupunctured liver hydatid caused extensive metastasis in hydatid abdominal cavity; the patient recovered after operation [62]. One diabetic patient without controlling blood glucose suffered from diabetic feet because of infection and recovered after hypoglycemic and anti-infection treatments.

### 3.9. Hemorrhage (Table 8)

Among 10 cases of acupuncture-induced local hemorrhage (Table 8), 8 patients recovered, 1 improved, but 1 died. The positions of hemorrhage included eyes (*n* = 2) [6, 66], extraperitoneal (1) [67], thyroid (1) [68], hypoglossus (2) [69, 70], suffocated death from hematoma compressed trachea (1) [71], hand (1) [72], 1 case of buttock hematoma due to acquired hemophilia B which improved after treatment [73], and lower limb (1) [74].

### 3.10. Complications Caused by Broken Needles (Table 9)

Seven cases of accidents due to broken or bent needles were identified (Table 9). Five recovered after the surgery [75–79], and 2 cases of bent needles were slowly pushed out by acupuncturists [6, 80].

### 3.11. Other Complications Associated with Acupuncture (Table 10)

A total of 15 other complications associated with acupuncture were reported (Table 10): aphonia [81], hoarseness [82], allergy to electroacupuncture [83] and metal [84], epilepsy [85, 86], fever [87], cough [88], thirst [88], infusion reaction [89], hyperventilation syndrome [90], and aggravation of fatigue [91]. Of them, 14 cases recovered completely and 1 improved.

One patient was not allergic after several acupuncture treatments, but systemic allergy occurred after EA treatment [65]. Among three patients with acupuncture-induced epilepsy, only one had a history of epilepsy [85, 86].

## 4. Discussion

The studies about safety of acupuncture are gradually increasing. One study protocol of a randomized controlled trial is efficacy and safety of acupuncture for chronic dizziness [92]. This trial's aim is to get result that acupuncture has good efficacy and without adverse effect for chronic dizziness. We hope it is success.

Some studies that researched acupuncture as an alternative means for pediatric diseases found that it is safe, feasible, and acceptable [93–99]. One study explored acupuncture as an effective therapy of pain relief for children and adolescents after tonsillectomy [100]. Severe throat pain can result from tonsillectomy and last up to 10 days in children. Codeine elixir has long been used for pain relief but has recently been banned by the Food and Drug Administration due to a recently recognized risk of death. This study suggested that acupuncture decreases perceived pain in children and adolescents after tonsillectomy. These data, combined with the cost effectiveness, safety, and ease of administering acupuncture, suggest that further studies exploring the effectiveness of acupuncture in juveniles after tonsillectomy are merited.

One research evaluated the feasibility of delivering acupuncture in an emergency department (ED) to patients presenting with pain and/or nausea [101]. The acupuncture group comprised 200 patients who received usual medical care and acupuncture; the usual care group comprised 200 patients with retrospective data closely matched from ED electronic health records. The results confirmed that acupuncture in the ED appears safe and acceptable for patients with pain and/or nausea. Further high-quality, sufficiently powered randomized studies evaluating the cost-effectiveness and efficacy of the add-on effect of acupuncture are recommended.

Some reports confirmed that acupuncture for pregnant women is safe and effective [102–109]. For example, one reported a complete recovery from Bell's palsy (BP) of a 27-year-old woman, 27-week pregnant, after 2 weeks of acupuncture treatment. Prior to treatment, her House-Brackmann facial nerve grading system (HBS) was II, Nottingham facial nerve grading system was 50.88%, and the Facial Disability Indexes (FDI) were 90. After 2 weeks, her symptoms had disappeared, her face was restored to normal, HBS was I, Nottingham was 96.46%, and FDIP was 100. These results suggest that acupuncture may be a safe, alternative treatment for BP in pregnancy [110]. Another study described patients' experience of acupuncture treatment in low back and pelvic pain during pregnancy. Women received acupuncture treatment from gestational week 20 or week 26, for a period of 6 weeks divided into eight sessions of 30 minutes each. The results of Pain-O-Meter and visual analogue scale (POM-VAS), Short-Form McGill Questionnaire (SF-MPQ), and Short-form-36 health survey (SF-36) showed a relief of pain. Telephone interviews confirmed that expectations of treatment were fulfilled. The authors suggested that it may be advantageous to begin acupuncture therapy later in pregnancy to maximize pain relief [111].

**Figure 2:**
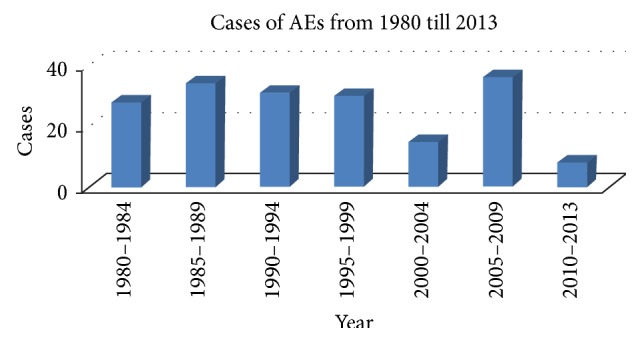
Distribution of cases of acupuncture-associated complications reported from 1980 to 2013.

However, complications and adverse effects in medical practice always concern the public and the medical profession. Acupuncture has been used for several thousand years in China. Although it has been deemed a safe and reliable therapy, the rare adverse effects and complications should arouse concerns. During the 33 years from 1980 to 2013, about 182 cases of acupuncture-associated complications and adverse effects were reported in China, including 25 fatal cases. The frequency of acupuncture associated complications reported in China appears to be steady over time (see Figure 2).

**Figure 3:**
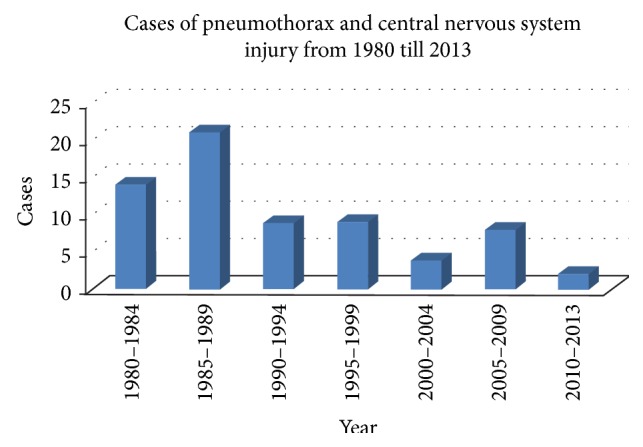
Distribution of cases of pneumothorax and central nervous system injury from 1980 to 2013.

As indicated in Table 1, the most frequent complication of acupuncture treatment is internal organ, tissue, or nerve injury. Of the 115 reported cases involving internal organ, tissue, or nerve injury, 30 (26.08%) were pneumothorax, 37 concerned the central nervous system (32.17%), others included injury in peripheral nerve, organ, and other tissues. Based on our research, one major cause of direct thrusted injuries to organ, tissue, or nerve is the lack of knowledge about anatomy and other systems. In 1980s, the acupuncturists or individuals in many country grassroot regions performed acupuncture because of low cost and convenience, but the deficient knowledge on anatomy led to many cases of pneumothorax and subarachnoid hemorrhage, as well as injuries to abdomen organs, heart, and peripheral nerves. With the increasing requirement for acupuncturists, these accidents decreased from the 1990s. Particularly, the frequency of pneumothorax and central nervous system injury appears to be on the decline since the 2000s (see Figure 3). This may be because the government has demanded that the acupuncturists should have licenses and formal education background if they practice in clinic in recent years. They should also undergo short time training every year. All these make the acupuncture technique become more and more standardized so that the accidents of pneumothorax and central nervous system injury are reduced. However, there is a possibility that we lack the accident reporting system so that the incidences were underreported.

We put forward suggestions for the medical system in order to avoid more accidental injury on organ, such as enhancing training on anatomy for acupuncturists; setting up more continuation courses on the safety of acupuncture practice for acupuncturists; establishing a reporting system on the incidents of acupuncture adverse effect; and safety courses and certificates should be required in order to obtain the license of acupuncture in China.

The acupuncturists (1) should avoid important organs and tissues during selection of acupoints and reposition if the patient changes body position; (2) do not distract attention during treatment and do not move the patient after acupuncture so as to avoid accidents; for unconscious patients unable to cooperate, shallow needling or not retaining needle is preferred, and the process of treatment should be strictly monitored; (3) inquire detailed medical history and carefully determine the needling depth for patients with emphysema or hemorrhagic disease. Moreover, traditional medicine holds that acupuncture should be performed to bring about the desired sensations of “sour, numb, heavy, and swelling.” Many acupuncturists and patients think that a stronger sensation of needling will bring about better therapeutic effects, but excessively violent operation will also cause accidents. The 2 cases of irritable stomach bleeding due to excessive irritation [35, 36] and the 1 case of femoral neck fracture due to myospasm [45] are typical examples and should alert clinicians. In case of suspected acupuncture-induced injury, the doctor should prolong the time of observation and warn for prompt treatment.

Syncope is also a common acupuncture-induced accident. The hungry, thirsty, drunken, or nervous patients should be asked to eat, drink, or rest for half an hour before treatment and calm down. They should be observed for a moment during and after treatment to avoid syncope. Once syncope occurs, needles should be pulled out immediately, sugar water should be provided, and the patient should lie down with head low; if the symptom becomes severe, take appropriate treatment. Two patients had severe adverse reaction like shock, with the clinical manifestation, including loss of consciousness, respiratory arrest, and carotid pulselessness. After doing CPR, both of them recovered [54, 55]. Reviewing medical history, one was found to have the similar experience several years ago [55]. Therefore, acupuncturists are required to inquire patients' medical history carefully and learn to deal with emergencies.

Acupuncture infection usually occurs in rural grassroot health centers with low awareness of hygiene, but accidents will be largely controlled along with the use of disposable needles and the popularization of health knowledge. Moreover, tetanus is still an adverse event that should be strictly prevented, and once it occurs, it will cause a high mortality rate. Along with the increasing incidence of diabetes, for patients with poorly controlled blood glucose [112], careful operation is required to avoid infection due to disunion of acupuncture-caused wounds.

The acupuncture-induced bleeding and hematoma are unavoidable; thus to reduce their incidence rates, acupuncturists should (1) get familiar with the anatomy of acupoints and avoid blood vessels during needle manipulation; (2) avoid manipulation methods such as lifting and thrusting when acupuncturing intraorbital acupoints; (3) appropriately extend the time of compression for patients with hypertension, arteriosclerosis, or inclination to hemorrhage and for women during the menstrual period. The acupuncture-induced hematoma is usually cold compressed within 24 hours and hot compressed after 24 hours. H. Liu and X. H. Liu [74] suggested pressing the local hematoma site heavily for a long time, which could immediately disperse the swelling, without leaving bruises. This method is recommended for other acupuncturists.

With a long history in China, acupuncture has been widely accepted and applied in people's daily life due to its exceptional therapeutic effects and low side effects. As early as 1980, WHO unveiled 43 kinds of diseases that can be treated with acupuncture. The number had increased to 107 in 2002 [113], from which we can see that acupuncture has been recognized by an increasing number of people and more research in this field is being undertaken. In countries where acupuncture is widely used, it is inevitable to encounter the occurrence of some side reactions in acupuncture therapies. However, the accident rate in acupuncture is relatively low. Although existing reports in China show no statistical data about acupuncture accidents, some studies conducted in large subject pools in Germany reveal some relevant information. It has been reported that Endres et al. [114] conducted a study about accidents in acupuncture therapies on 190,924 patients. The study showed an occurrence of 14,449 acupuncture accidents, which accounted for 7.57% of the total subject pool. According to statistics conducted by Witt et al. [115] on 229,230 clinical acupuncture cases, there were 19,726 accidents, which occupied 8.6% of the total subject pool. Chinese literatures show that most of the acupuncture accidents are caused by acupuncturist's lack of corresponding techniques and nonstandard operations. Since 2005, the Chinese National Administration of Quality Supervision, Inspection and Quarantine and the Chinese National Standardization Management Committee have issued a total of 18 acupuncture technical operation specifications in two batches, including terms and definitions, operating procedures and requirements, operating methods, attentions, and contraindications. Among them, operating procedures and requirements specifically include the selection of needles, acupuncture points and acupuncture positions, environmental requirements, the disinfection of needles, selected acupuncture points and acupuncturist's hands, specific operation techniques, and after-treatments of wounds [116]. The acupuncture technical operation specification series covers a wide area and contains comprehensive and specific contents, but it still shows some deficiency in the popularization and implementation of acupuncture.

In conclusion, we recommend that the qualifying training of acupuncturists should be systemized and the clinical acupuncture operations be standardized in order to effectively prevent the occurrence of acupuncture accidents, enhance the influence of acupuncture, and further popularize acupuncture to the rest of the world. All this would mean a huge step towards modernization of acupuncture.

## Figures and Tables

**Table 1:** Pneumothorax associated with acupuncture.

Author/year (reference)	Cases	Age/sex	Disease treated	Punctured site	Practitioner	Follow-up
Jiang, 1980 [117]	1	54/F	Gastroptosis	RN15	Not specified	Recovered
Cai and Wang, 1982 [118]	1	41/M	Numbness and pain of shoulders and chest	Shoulder and back	Not specified	Recovered (1 mo)
Zheng and Pang, 1983 [119]	1	21/M	Stiff neck	GB21	Not specified	Recovered after surgery (1 mo)
Gao, 1984 [120]	1	50/F	Chest and back pain	Back	Factory doctor	Recovered (12 d)
Duan and Wang, 1984 [29]	2	26/F	Bronchitis	GB21, BL13, EX-B2	Not specified	Recovered (3 d)
		50/F	Bronchitis	GB21, BL13	Not specified	Recovered (7 d)
Chang, 1984 [121]	1	33/M	Back pain	Back	Country doctor	Recovered (19 d)
Yan, 1985 [122]	1	55/F	Chronic bronchitis	RN22	Health center	Recovered (16 d)
Hu, 1986 [123]	1	58/F	Pulmonary heart disease	BL13	Not specified	Recovered (13 d)
Zhang, 1986 [124]	1	52/M	Cervical pain	Left shoulder	Factory doctor	Recovered (20 d)
Yang, 1986 [6]	1	57/F	Chronic asthmatic disease	BL12	Not specified	Dead
Jin, 1987 [125]	1	26/F	Shoulder pain	SI13	Acupuncturist	Recovered (9 d)
Song and Xu, 1987 [126]	1	60/F	Shoulder pain	GB21	Not specified	Not specified
Ruan et al., 1992 [127]	1	35/F	Hysteria	Supraclavicular fossa	Acupuncturist	Recovered (10 d)
Zhang et al., 1992 [38]	3	53/F	Chronic bronchitis and emphysema	BL18	Not specified	Recovered (1 wk)
		65/F	Cough	RN22		Recovered (1 mo)
		21/M	Spasmodic torticollis	LI17		Recovered (2 wk)
Lu, 1993 [128]	1	60/M	Cough, chest, and back pain	Back	Acupuncturist	Recovered (1 wk)
Xia, 1993 [129]	1	60/M	Back pain caused by hyperplastic spondylitis	BL18, BL23	Acupuncturist	Recovered (10 d)
Li and Chen, 1997 [130]	1	65/F	Shoulder pain	Scapular region	Clinic	Recovered (5 d)
Ma and Zhang, 1997 [131]	1	48/M	Neck and shoulder pain	Shoulder and back	Clinic	Recovered (1 wk)
Wang, 1999 [7]	1	47/F	Shoulder and leg pain	LI17	Not specified	Dead
Song and Wu, 2001 [9]	1	45/F	Scapulohumeral periarthritis	GB21, SI11	Hospital	Recovered (1 wk)
Qin and Ao, 2003 [132]	1	55/M	Intercostal neuralgia	The sixth intercostal space on the anterior axillary line	Factory doctor	Recovered (2 wk)
Zha, 2006 [133]	1	32/M	Chronic hepatitis B	Chest and back	Itinerant doctor	Recovered (14 d)
Gan et al., 2006 [134]	1	30/M	Neck and back pain	Back	Individual clinic	Not specified
Huo et al., 2007 [135]	1	39/M	Chest and back pain	Not specified	Not specified	Recovered after surgery (7 d)
Liu, 2007 [136]	1	50/M	Cervical and back pain	Back	Individual clinic	Not specified
Ma, 2007 [137]	1	35/F	Shoulder pain	Not specified	Individual clinic	Recovered
Zhang and Zhao, 2012 [138]	1	65/F	Cervical spondylopathy	GB21	Acupuncturist	Recovered (10 d)

**Table 2:** Central nervous system injury associated with acupuncture.

Author/Year (reference)	Cases	Age/Sex	Disease treated	Punctured site	Complication	Onset after acupuncture	Follow-up
Liu, 1980 [139]	1	19/F	Impaired vision	GB20	Subarachnoid hemorrhage	1 h	Recovered (20 d)
Liu, 1981 [14]	3	16/M	Schizophrenia	DU16	Subdural hematoma	Immediately	Dead
		30/F		DU14	Cervical and thoracic cord injury		
		30/M		GB20	Subarachnoid hemorrhage	1 h	Recovered (47 d)
Bao and Gao, 1983 [140]	3	30/F	Eyelid muscle twitch	GB20	Subarachnoid hemorrhage	Immediately	Recovered (14 d)
		27/M	Bulbar palsy	DU15			Recovered (20 d)
		40/F	Headache	GB20			Recovered (19 d)
Chen and Wu, 1985 [141]	1	30/F	Hysteria	Ex-HN18	Subarachnoid hemorrhage and medulla oblongata hemorrhage	1 d	Dead
Yang et al., 1985 [8]	1	15/M	Chronic tracheitis	Between 2 and 3 thoracic spinous process	Subarachnoid hemorrhage	Immediately	Recovered (1 mo)
Chen and Huang, 1985 [142]	1	57/M	Facial Spasm	Neck	Subarachnoid hemorrhage	Immediately	Recovered (3 wk)
Su et al., 1985 [143]	1	11/F	Deaf-mute	DU15	Subarachnoid hemorrhage	At night	Recovered (1 wk)
Yu, 1986 [144]	7	42/M	Psychosis	DU15	Subarachnoid hemorrhage	Several hours	Recovered (1 mo)
		4.5/M	Cerebral agenesis with aphasia	DU15		Immediately	Recovered (20 d)
		29/M	Weakness of limbs	DU15, DU16		Minutes after treatment	Recovered (1 mo)
		22/M	Acid swells of the neck	Back neck		Immediately	Recovered (40 d)
		55/F	Aural vertigo	Back neck			Recovered (20 d)
		24/F	Stuffy head	GB20			Recovered (1 mo)
		22/M	Facial paralysis	Ex-HN21			Recovered
Chen, 1987 [145]	1	37/F	Neck pain	DU15	Subarachnoid hemorrhage	1 min	Recovered (1 mo)
Jiang and Chen, 1987 [146]	1	77/F	Stiff neck	GB20	The cerebellopontine and subarachnoid hemorrhage	After treatment	Dead
Zhou, 1988 [147]	1	15/M	Cold	GB20	Subarachnoid hemorrhage	During the treatment	Dead
Mi et al., 1989 [148]	1	28/F	Neurosis	DU15	Subarachnoid hemorrhage	2 d	Recovered (27 d)
Wu and Xu, 1990 [149]	1	57/M	Stroke	Ashi points near C3	Subarachnoid hemorrhage	1 h	Dead (10 d)
Liu, 1992 [150]	1	28/M	Insomnia	GB20	Acute subdural hematoma	3 hours	Recovered after surgery
Mi, 1993 [12]	1	73/M	Cerebral hemorrhage	LI11, LI4, GB30, ST36, GB39, GB14, ST2	Cerebral hemorrhage reformulation	10 min	Dead
Jiang et al., 1996 [151]	2	45/M	Cervical spondylopathy	GB20	Subarachnoid hemorrhage	Immediately	Recovered (30 d)
		54/M		Neck			Recovered (1 mo)
Liu et al., 1996 [152]	1	35/M	Ankylosing Spondylitis	DU16	Medulla oblongata hemorrhage	5 hours	Dead
Bian et al., 1997 [153]	1	29/F	Headache caused by pesticide poisoning	GB20	Subarachnoid hemorrhage	3 min	Recovered (1 mo)
Wang, 1999 [10]	1	54/M	Low back pain	BL37	Acute subarachnoid hemorrhage	Immediately	Recovered
Wang, 1999 [154]	1	39/F	Neurosis	GB20	Cervical cord injury	Immediately	Dead
Song and Wu, 2001 [9]	1	58/M	Hypertensive cerebral hemorrhage	KI01	Cerebral hemorrhage reformulation	Immediately	Dead
Li et al., 2003 [155]	1	55/M	Neck and back pain	Neck and back	Cervical spinal epidural hematoma	Immediately	Recovered
Niu and Zhang, 2006 [156]	1	42/M	Headache	Neck	Cisterna magna hemorrhage	Not specified	Recovered after surgery
Li et al., 2008 [157]	1	36/F	Migraine	DU16	Subarachnoid hemorrhage	At night	Recovered (3 wk)
Chen, 2009 [11]	1	43/F	Lumbago pain	Waist	Subarachnoid hemorrhage	Immediately	Recovered (1 mo)
Li et al., 2011 [13]	1	45/F	Toothache	RN16, RN10, RN9, RN4	Leukemia acute intracerebral hemorrhage	Immediately	Dead (2 d)

**Table 3:** Peripheral nerve injury.

Author/Year (reference)	Cases	Age/Sex	Disease treated	Punctured site	Complication	Practitioner	Follow-up
Tang and Fang, 1986 [15]	1	Not specified	Facial paralysis	Not specified	Aggravated facial paralysis	Not specified	Recovered
Nie and Zhou, 1990 [16]	1	26/M	Left eye pain	SJ17	Cardiac arrest	Acupuncturist	Recovered (1 min)
Yan, 1994 [17]	1	57/M	Cervical dislocation	Bitong point	Phrenic nerve injury	Acupuncturist	Recovered (1 wk)
Yang and Wang, 1996 [18]	1	62/M	Lateral rectus paralysis	Ex-HN07	Optic atrophy	Acupuncturist	Blind
Xu and Liu, 1997 [19]	1	48/M	Tinnitus and hearing loss	ST1, ST2, BL2, LI20, SJ21, SI19	Oculomotorius injury	Not specified	Recovered (17 d)
Huang and Wei, 1997 [20]	1	53/F	Trigeminal nerve pain	Around the mandible	Trigeminal nerve injury	Acupuncturist in the stomatological hospital	Improved (3 mo)
Wang, 1999 [10]	1	54/F	Lumbago	GB30	Sciatic nerve injury	Private practitioner	Unrecovered
Ruan et al., 2009 [21]	1	67/F	Lumbago	GB34	Peroneus nerve injury	Not specified	Recovered (3 wk)

**Table 4:** Organ injury associated with acupuncture.

Author/year (reference)	Cases	Age/sex	Disease treated	Punctured site	Complication	Follow-up
Liu, 1981 [14]	1	19/F	Schizophrenia	RN15	Cardiac tamponade	Dead
Zhu, 1990 [22]	1	64/M	Chest stiffness and rib pain	LR14	Aortoclasia	Dead
Shi, 1993 [23]	1	42/F	Witchcraft	Limbs, chest, abdomen, back	Aortoclasia	Dead
Xie and Lin, 2003 [24]	1	35/F	Intercostal neuralgia	Right breast	Cardiac trauma	Dead
Luo et al., 2006 [25]	1	40/M	Chronic esophagitis	RN15	Cardiac tamponade	Recovered
Zhang and Dong, 2006 [26]	1	37/M	Chest tightness, asthma	Chest and back	Cardiac tamponade	Dead
Zhu et al., 2008 [27]	1	44/F	Diabetes	Chest	Cardiac tamponade	Dead
Yang, 1991 [28]	1	21/M	Bad cold	BL13	Chylothorax	Recovered (2 wk)
Zheng and Zhao, 1983 [30]	1	31/F	Stomachache	ST25, LR14, RN12	Gallbladder perforation and biliary peritonitis	Recovered after surgery
	1	59/M	Cervical spondylopathy	RN12, RN13, ST25		
Deng, 1985 [31]	1	45/F	Acute attack of chronic cholecystitis	ST21	Gallbladder perforation	Not specified
Bai, 1991 [32]	1	32/F	Paralytic ileus	Upper abdomen	Gallbladder perforation and biliary peritonitis	Recovered after surgery
Duan and Wang, 1984 [29]	1	42/M	Intestinal spasm	RN8, RN12, ST25	Intestinal perforation and suppurative peritonitis	Recovered after surgery (14 d)
Zhang, 1997 [33]	4	51/M	Paroxysmal abdominal pain	Abdomen	Localized peritonitis	Recovered after surgery
		47/F	Right lower abdominal pain			
		53/F	Abdominal pain, diarrhea			
		37/F	Periumbilical pain			
Xiao, 1985 [34]	1	20/M	Abdominal discomfort	RN12	Gastric perforation	Recovered after surgery (11 d)
Huang, 1999 [35]	1	54/M	Gastric ulcer	ST36	Gastric ulcer with perforation	Recovered after surgery (10 d)
Tang et al., 2006 [36]	1	61/M	RA	ST34, ST40, SP6, SP10, GB33, GB34	Gastric stress ulcer and hemorrhagic shock	Recovered (2 mo)
Liu et al., 1992 [37]	1	2/M	Diarrhea	RN12, ST25, DU1	Complete intestinal obstruction	Recovered after surgery
Zhang et al., 1992 [38]	1	45/F	Chronic bronchitis, coronary disease	GB21, BL13, BL23	Multiple organ injury	Dead

**Table 5:** Other tissue injury.

Author/year (reference)	Cases	Age/sex	Disease treated	Punctured site	Complication	Practitioner	Follow-up
Chen, 1980 [39]	1	33/F	Sore throat	Neck	Cervical common carotid aneurysm	Not specified	Recovered after surgery (3 mo)
Wang, 1987 [40]	1	42/F	Thyroid cancer (anaesthesia for thyroidectomy)	SJ17	Sinus caroticus syndrome, shock	Not specified	Recovered
Zhang et al., 1992 [38]	1	39/M	Asthma	BL13	Pleural shock	Not specified	Recovered (2 d)
Zhu, 1986 [41]	1	56/M	Chest distress	RN22	Asphyxia	Acupuncturist	Dead
Gao, 1989 [42]	1	73/M	Cough	LI18	Dyspnea	Not specified	Recovered (3 d)
Liu et al., 1988 [43]	1	53/M	Blepharospasm	The lateral upper eyelid	Retinal detachment	Health worker	Recovered after surgery
Yang and Wang, 1996 [18]	1	63/F	Left eye ptosis	Periocular	Traumatic cataract	Acupuncturist	Blind
Wang, 1982 [44]	2	20/M	Traumatic mydriasis	EX-HN5	The orbicularis oculi muscle tremor	Not specified	Recovered (3 d)
		35/F	Right migraine		Speech and swallowing difficulties		Recovered (2 h)
Li and Zhou, 1980 [45]	1	40/M	Schizophrenia	EX-HN5	Femoral neck fracture	Acupuncturist	Not specified
Liang and Song, 1984 [46]	1	23/M	Cramp	BL57	Flexor hallucis muscle and digitorum longus muscle scar contracture	Health worker	Recovered after surgery
Chen, 1992 [47]	1	17/F	Shortsightedness	GB20, EX-HN17	Nape spasm	Acupuncturist	Recovered (6 d)
Wang, 1994 [48]	1	65/F	Stroke	LI4, SJ5, LI15	Subluxation of wrist	Intern	Recovered (1 wk)
Shi and Chen, 1994 [49]	1	52/M	Facioplegia	LI11	Elbow pain	Acupuncturist	Recovered (2wk)
	1	61/F	Scapulohumeral periarthritis	Not specified	Shoulder pain		Recovered (20 d)
Wang, 1999 [10]	1	53/M	Cough, epistaxis	LU6	Amyotrophy	Private practitioner	Unrecovered
Luo and Huang, 2006 [50]	2	42/M	Amyotrophic lateral sclerosis	Upper limb	Fasciculation	Not specified	Improved
		63/M		Upper limb			Improved

**Table 6:** Syncope associated with acupuncture.

Author/year (reference)	Cases	Age/sex	Disease treated	Punctured sites	Position	Start time	Causes	Practitioner	Follow-up
Yang, 1986 [6]	1	32/M	Facioplegia	GB14, ST 6, ST4, SJ 17, LI20, LI4	Sitting	5 min after inserting needle	Limosis	Acupuncturist	Recovered
Shao, 1989 [51]	1	53/M	Myotenositis of long head of biceps brachii	LI15, LI11, ashi	Not specified	After inserting needle	After drinking	Acupuncturist	Recovered (20 m)
Shi and Chen, 1994 [49]	1	57/F	Stomachache	LI3, ST 36, PC6	Not specified	Finish needling	Limosis	Acupuncturist	Recovered (30 min)
Guo, 1995 [158]	2	65/F	Scapulohumeral periarthritis	Ex-UE01, GB21, LI14, LI11, SJ5, LI4	Clinostatism	Finish needling	Weakness	Acupuncturist	Recovered
		24/F	Waist sprain	BL40, ashi point and acupoint of bladder meridian		Finish needling	Nervous	Acupuncturist	Recovered (10 min)
Wu et al., 2001 [56]	1	24/F	Insomnia	PC6, ST 36, HT7	Not specified	At night	Not specified	Acupuncturist	Recovered (2 d)
Liu, 2001 [52]	3	45/F	Lumbago pain	BL23, BL40, DU3, GB30, KI7	Not specified	Finish needling	After drinking	Acupuncturist	Recovered
		34/F	Shoulder pain	LI11, LI14, LI15, SJ5, SJ14	Sitting	After inserting needle	Tiredness	Acupuncturist	
		56/F	Right thumb pain	LI4, LI5, LI11, ashi point	Not specified	On the way home	Limosis	Acupuncturist	
Ma, 2005 [54]	1	28/M	Prosopalgia	EX-HN5, LI4, ST6, SJ5	Sitting	5 min after inserting needle	Not specified	Acupuncturist	Recovered (30 s)
Long et al., 2006 [55]	3	72/M	Stroke	LI11, SJ5, ST36, SP6, EX-UE17, EX-LE11	Clinostatism	10 min after inserting needle	Nervous	Acupuncturist	Recovered (2 min)
		41/F	Cervical spondylopathy	BL10, GB20	Sitting	1 min after inserting needle	Not specified	Acupuncturist	Recovered (30 min)
		42/F	Lumbago pain	ST36	Clinostatism	After inserting needle	Heavy stimulus	Acupuncturist	Recovered (2 min)
Liu, 2007 [159]	1	42/F	Scapulohumeral periarthritis	Ex-UE01, LI14, Ashi point, SJ3	Sitting	10 min after inserting needle	Not specified	Acupuncturist	Recovered (30 min)
Chen, 2009 [11]	1	42/M	Acute lumbar sprain	EX-B5, BL40	Not specified	Finish needling	Not specified	Acupuncturist	Recovered (10 min)
Liao and Guo, 2009 [160]	1	57/F	Gouty arthritis	GB20	Clinostatism	After inserting needle	Limosis	Acupuncturist	Recovered (10 min)
Li et al., 2009 [53]	2	48/M	Cervical spondylopathy radiculaire	GB21, LI11, SJ5, Ex-B5	Not specified	6 h after treatment	Not specified	Acupuncturist	Recovered after stop treatment
		68/F	Facial paralysis	GB14, ST2, ST4, ST6, SJ17, LI4, Ex-HN16		12 h after treatment	Not specified	Acupuncturist	Recovered after stop treatment

**Table 7:** Infection associated with acupuncture.

Author/year (reference)	Cases	Age/sex	Disease treated	Punctured site	Diagnosis	Practitioner	Follow-up
Zhang, 1980 [161]	1	5/F	Heat, cough	Ex-UE19	Infection	Not specified	Middle finger disability
Xie and Zong, 1983 [62]	1	38/F	Right upper abdominal mass and discomfort	Ashi points	Abdominal metastatic hepatic hydatid	Local hospital	Recovered after surgery
Gao and Qi, 1989 [65]	1	54/M	Right leg pain	Local points	Third-degree burns with infection	Clinic	Not specified
Xu, 1990 [162]	1	19/M	Migraine	EX-HN5, GB14, DU20	Head abscess, intracranial infection	Health worker in the army	Recovered
Xia, 1993 [129]	1	37/F	Facioplegia	ST5	Right cheek hematoma with infection	Not specified	Recovered (1 wk)
Chen and Gao, 1995 [63]	1	30/M	Insomnia	Head	Encephalopyosis	Not specified	Recovered after surgery
Zhou, 1999 [64]	3	From 22 to 28/M (1); F (2)	Lumbar muscle strain	Loin	Tuberculous abscess on the body surface	Not specified	Recovered (3–6 mo)
Song and Wu, 2001 [9]	1	38/M	Rheumatic arthritis	EX-LE4, EX-LE5, GB34, SP9	Pyogenic arthritis	Individual clinic in the country	Recovered (2 wk)
Ding et al., 2008 [112]	1	60/F	Scapulohumeral periarthritis	ST38	Diabetes foot	Not specified	Remission after 3 days
Yang et al., 1990 [57]	1	2/F	Malnutritional stagnation	Ex-UE19	Tetanus	Illegal treatment	Dead
Liu, 1991 [58]	1	52/F	Headache	Ex-HN05, DU20, GB20, LI4	Tetanus	Village acupuncturist	Recovered (2 wk)
Liu, 1992 [59]	1	52/F	Leg pain	Not specified	Tetanus	Private practitioner	Recovered (1 mo)
Sun and Hu, 1997 [60]	1	23/M	Facioplegia	Not specified	Tetanus	Health-center	Remission after 3 days
Wang, 1999 [10]	1	60/M	Headache and fever	GB20, GB21, SJ5	Tetanus	Not specified	Dead
Chen et al., 2008 [61]	1	62/F	RA	Knee	Tetanus	Illegal treatment	Dead

**Table 8:** Hemorrhage.

Author/year (reference)	Cases	Age/sex	Disease treated	Punctured site	Complication	Practitioner	Follow-up
Yang, 1986 [6]	1	28/F	Chronic conjunctivitis	BL1	Eye hematoma	Acupuncturist	Recovered (14 f)
Li, 1989 [67]	1	62/F	Teratoma of ovary	Hypogastrium	Extraperitoneal hematoma	Roving doctor	Recovered after surgery
Cai, 1991 [68]	1	47/M	Neck mass	Neck	Thyroid intracapsular hemorrhage with apnea	Not specified	Recovered after surgery (10 d)
Han, 1994 [69]	1	56/M	Stroke	RN23	Sublingual hematoma	Not specified	Recovered (1 wk)
Zeng and Liu, 1996 [71]	1	50/M	Cough	ST9	Hematoma compression tracheal cause apnea	Unauthorized acupuncturist	Dead
Wang, 1996 [70]	1	72/M	Cerebral infarction	Ex-HN20	Sublingual hematoma	Not specified	Recovered (2 d)
Gan, 2000 [66]	1	46/F	Cold headache	Ex-HN05	Orbital hemorrhage	Not specified	Recovered (1 mo)
Jiang, 2001 [72]	1	68/M	Hypertension, stroke	LI4	Hand hematoma	Acupuncturist	Recovered (7 d)
Duan, 2007 [73]	1	65/F	Neck, waist, and leg pain	Hip	Buttock hematoma	Not specified	Improved
H. Liu and X. H. Liu, 2007 [74]	1	61/M	Cerebral thrombosis	SP6	Lower extremity hematoma	Acupuncturist	Recovered (1 d)

**Table 9:** Complications caused by broken needles.

Author/year (reference)	Cases	Age/sex	Disease treated	Punctured site	Complication	Practitioner	Follow-up
Yang, 1986 [6]	1	45/M	Flaccid paralysis	ST36, LI11	Bent needle	Acupuncturist	Recovered
Lu and Teng, 1994 [75]	1	39/M	Scapulohumeral periarthritis	Supraclavicular fossa	Hemopneumothorax caused by broken needle	Country doctor	Recovered after surgery
Wang, 2000 [80]	1	54/M	The left upper limb dysfunction	Upperlimb	Sticking of needle	Not specified	Recovered
Geng, 2005 [76]	1	58/M	Chronic bronchitis and emphysema	LU1	Damage of arteria coronaria and cardiac tamponade caused by embedded needle	Self	Recovered after surgery
Quan, 2008 [77]	1	43/F	Gastric disease	Xiphoid	Palpitation and paroxysmal pricking pain caused by broken needle	Not specified	Recovered after surgery
Liu and Yu, 2010 [78]	1	45/F	Multiple injuries by traffic	RN23	Pulmonary bulla caused by embedded needle	Not specified	Recovered after surgery
Cheng, 2010 [79]	1	55/M	Lumbago	Lower limb	Broken needle	Not specified	Recovered after surgery

**Table 10:** Other complications associated with acupuncture.

Author/year (reference)	Cases	Age/sex	Disease treated	Acupoint	Complication	Practitioner	Follow-up
Wang and Lan, 1980 [81]	2	46/F	Intercostal neuralgia	PC6	Aphonia	Not specified	Recovered (3 d)
		36/F	Obstinate hiccup				
Zhou et al., 2005 [82]	1	43/F	Neck pain	Ex-B05	Hoarseness	Acupuncturist	Recovered (10 min)
Peng, 1982 [83]	1	54/not specified	Scapulohumeral periarthritis	Ex-UE01, GB21, LI11, SJ5	Allergy to electroacupuncture	Acupuncturist	Recovered (10 min)
Gao and Zheng, 2008 [84]	2	72/M	Nerve root cervical spondylopathy	EX-B2	Allergy to metal	Not specified	Recovered (1 wk)
		49/F	Cervical type cervical spondylopathy	EX-B2			Recovered (5 d)
Wang, 2004 [85]	1	35/M	Soft tissue injury	Ashi point	Epilepsy	Acupuncturist	Recovered
Dai, 2012 [86]	2	45/M	Epilepsy	Not specified	Epilepsy	Acupuncturist	Recovered (2 min)
		53/M	Cervical spondylosis				Recovered (1 min)
Li, 2000 [87]	1	52/M	Cerebral concussion	DU20, GB20, GB30, GB39, LI4, LI11, ST36	Fever	Acupuncturist	Recovered
Shang, 2006 [88]	2	65/F	Facial neuritis	GB14, BL2, ST2, SI18, RN24, LI4, ST36, LR3	Cough	Acupuncturist	Recovered (2 min)
		46/F	Obesity	ST25, ST36, SP15, RN6, LI11, SJ6, SP9, ST40	Thirsty	Acupuncturist	Recovered
Quan and Jiang, 2008 [89]	1	45/F	RA	Local points	Infusion reaction	Acupuncturist	Recovered (2 h)
Fang, 2010 [90]	1	35/F	Cervical pain	GB20, EX-B2	Hyperventilation syndrome	Acupuncturist	Recovered (15 min)
Wang, 2010 [91]	1	46/F	Nasopharyngeal carcinoma radiation sequela with fatigue	LI11, LI4, ST36, SP6, KI3; LU7, SI6, SJ3	Aggravation of fatigue	Acupuncturist	Improved
